# Accurate and Efficient Description of Acidic Zeolites with Plane‐Wave Density Functional Theory Using Range‐Separated Hybrid Functionals

**DOI:** 10.1002/cphc.202500147

**Published:** 2025-06-30

**Authors:** Philipp Huber, Philipp N. Plessow

**Affiliations:** ^1^ Institute of Catalysis Research and Technology Karlsruhe Institute of Technology (KIT) Hermann‐von‐Helmholtz Platz 1 76344 Eggenstein‐Leopoldshafen Germany

**Keywords:** benchmark, catalysis, density functional theory, theory, zeolite

## Abstract

Brønsted acidic zeolites and their reactivity are routinely studied computationally, mainly with periodic density functional theory (DFT) using the generalized gradient approximation (GGA). In many cases, large errors are observed at the GGA‐level of theory, in particular reaction barriers are often underestimated and the stability of carbocations is overestimated. The use of ab initio methods, such as MP2 and CCSD(T), also with local approximations, is mostly limited to nonperiodic cluster models. In this work, for a set of reaction energies and barriers, the random phase approximation and common density functionals are investigated by comparison to DLPNO‐CCSD(T) and complete basis set extrapolated MP2 calculations on large cluster models. The most accurate functionals are the range‐separated hybrids *ω*B97M‐D4, *ω*B97M‐V, *ω*B97X‐D4, *ω*B97X‐V, and *ω*B97‐D. Compared to our reference calculations, these functionals give mean absolute errors below 8 kJ mol^−1^ and also lead to few outliers. *ω*B97M‐D4 performs best, with an MAE of 5.1 kJ mol^−1^ and an error that is smaller than that of complete basis set extrapolated MP2. Range‐separated functionals are shown to work well in periodic calculations with plane‐wave DFT. This allows the efficient calculations of very accurate reaction energies and barriers directly for the periodic system at modest computational cost.

## Graphical abstract

Range‐separated functionals, such as *ω*B97M‐D4, are shown to perform very well for reactions in acidic zeolites. Importantly, they work well with plane‐wave density functional theory and can thus be directly applied to periodic models at modest computational cost.

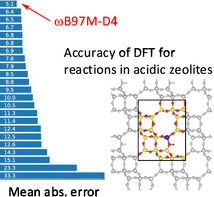

## 1 Introduction

Zeolites are used as molecular sieves and also as catalysts in many applications. Brønsted acidic zeolites, where a silicon atom of the SiO_2_ framework is substituted by Al, are studied routinely with density functional theory (DFT) and ab initio methods, in particular with respect to application in the methanol‐to‐olefins (MTO) process.^[^ ^1^, ^2^, ^3^, ^4^, ^5^, ^6^ ^]^

The reactivity of acidic zeolites has been studied theoretically for decades,^[^ ^5^, ^6^, ^7^, ^8^ ^]^ with early studies using very small cluster models due to the technical limitations at that time.^[^ ^9^ ^]^ Later, periodic DFT calculations have been introduced for zeolite calculations and are now the standard approach in zeolite catalysis, typically in combination with the very efficient PBE‐D2 or PBE‐D3 approach, that is, dispersion corrected DFT at the generalized‐gradient‐approximation (GGA) level of theory.^[^ ^5^, ^6^ ^]^ Nevertheless, large cluster models are also being used for routine investigations, often in combination with accurate hybrid functions, such as *ω*B97‐D.^[^ ^10^, ^11^, ^12^ ^]^

Highly accurate calculations employing both ab initio methods and realistic model systems have been pioneered in the group of Joachim Sauer.^[^ ^13^ ^]^ Here, periodic calculations were combined with cluster models, which allowed the application of ab initio methods, mostly MP2 but for small cluster models also canonical CCSD(T).^[^ ^13^, ^14^, ^15^, ^16^, ^17^, ^18^ ^]^ Later, approximate coupled cluster methods such as DLPNO‐CCSD(T) were additionally employed.^[^ ^18^, ^19^ ^]^ The focus of the Sauer group has been to perform accurate calculations rather than to benchmark density functionals. Nevertheless, as a byproduct, the early calculations revealed that the dispersion lacking in GGA‐functionals such as the Perdew Burke Ernzerhof (PBE) functional is crucial to compute adsorption energies.^[^ ^14^, ^15^ ^]^ With the introduction of Grimme's dispersion correction (D,^[^ ^20^ ^]^ D2,^[^ ^21^ ^]^ D3,^[^ ^22^ ^]^ and D4^[^ ^23^ ^]^), adsorption energies were improved, however, Sauer and coworkers have found that PBE‐D2 slightly overbinds adsorption reactions in zeolites in many cases.^[^ ^13^ ^]^ For methanol and ethanol adsorption in H‐MFI, PBE‐D2 was found to give binding energies that are too strong by 19 and 22 kJ mol^−1^, respectively.^[^ ^17^ ^]^ For different propene, butene, and pentene isomers, PBE‐D2 binding energies in the form of the *π*‐complex were found to be too strong by 18‐24 kJ mol^−1^.^[^ ^24^ ^]^ PBE and most nonhybrid functionals generally tend to underestimate barriers, and this is also true for zeolite‐catalyzed reactions.^[^ ^14^, ^15^ ^]^ In the case of zeolites, the relevant apparent activation energies (rather than intrinsic ones) often have to be computed with respect to a reactant in the gas phase. Apparent activation barrier will then contain the errors of both adsorption and intrinsic barrier. In the common case that a functional such as PBE‐D3 underestimates the barrier and overestimates the strength of adsorption, both errors will add up in the same direction to give a too low apparent activation energy.

An early work targeting specifically the benchmarking of density functionals in zeolites was published by Truhlar, who found the M06 and M06‐L functionals to be superior to PBE for adsorption.^[^ ^25^ ^]^ In our group, we were particularly interested in reaction barriers and looked at different types, ranging from low barriers for methylation reactions to high barrier for the initiation of the MTO process, that involve (de)hydrogenation. These high barriers in particular are problematic for most density functionals, where dispersion corrected PBE gives errors of 50 kJ mol^−1^ and larger.^[^ ^26^ ^]^ Errors of comparable size were found by Berger and Sauer for cracking reactions.^[^ ^19^ ^]^

Besides MP2 and CCSD(T), an ab initio approach that has gained popularity is the random phase approximation (RPA).^[^ ^27^, ^28^, ^29^ ^]^ In contrast to CCSD(T), the RPA is a method that is being used frequently not only in nonperiodic codes with atom‐centered basis functions but also in periodic plane‐wave programs. The RPA has been applied to study adsorption behavior^[^ ^30^ ^]^ and was found to be in good agreement with CCSD(T), typically underbinding but often within chemical accuracy ≈5 kJ mol^−1^).^[^ ^31^ ^]^ The RPA has, in combination with machine learning, also been applied recently in molecular dynamics (MD) simulations to determine adsorption enthalpies and barriers for olefin isomerization and cracking and was found to be in good agreement with experimental results.^[^ ^32^, ^33^, ^34^ ^]^ Van Speybroeck and coworkers have also combined the RPA with machine learning in MD simulations to study adsorbed isobutene and its equilibrium with the tert‐butyl cation.^[^ ^35^ ^]^ The nature of adsorbed isobutene is an interesting case^[^ ^36^, ^37^, ^38^, ^39^, ^40^ ^]^ as it suffers from the inaccuracies of nonhybrid DFT, which overstabilizes the cation in addition to the inaccuracies of the harmonic approximation, which underestimates the entropy of weakly bound species, such as the cation and the *π*‐complex.

Most of the approaches that were used to obtain highly accurate energies for zeolites are not ideal for routine applications. The construction of cluster models is not unique, somewhat cumbersome, and also not transferrable, for example, if one wants to study a different T‐sites. Periodic models are ideal, due to their computational simplicity and because they intrinsically capture all long‐range interactions. However, CCSD(T) is not applicable, and even RPA‐calculations are not affordable for routine applications for large unit cells such as H‐ZSM‐5. In our view, the ideal solution for routine calculations on many zeolite structures would be a periodic calculation with an accurate density functional. Generally, the most accurate available density functionals for thermochemistry, such as M06 or *ω*B97M‐V, are meta‐generalized‐gradient‐approximation (MGGA)‐hybrid functionals and are not routinely available in plane‐wave codes. This is changing, and MGGA‐hybrid functional calculations can be performed in the Vienna Ab Initio Simulation Package (VASP) since version 6.4.1.^[^ ^41^, ^42^ ^]^ Furthermore, linking against the library libxc^[^ ^43^ ^]^ allows access to many functionals.

In this work, we study several hybrid functionals, with an emphasis on those available in periodic calculations. Additionally, we investigate how well the RPA performs not only for adsorption, but also for reaction barriers in acidic zeolites.

## 2 Computational Details

Table S1 in the Supporting Information provides a detailed list of settings employed for each method. All plane‐wave calculations were performed using the projector augmented wave method (PAW)^[^ ^44^ ^]^ with VASP,^[^ ^41^, ^42^ ^]^ initially with version 5.4.1. To perform MGGA‐hybrid calculations, VASP 6.4.1 was employed, linked against libxc,^[^ ^43^ ^]^ version 5.2.2. Most calculations were performed using the standard PAW potentials (version 54), a cutoff of 400 eV for the expansion of the bands in plane waves, a plane‐wave basis set for the electronic density, which uses reciprocal lattice vectors with a norm up to 3/2 times larger than for the wave function, $\left|G_{\text{cut}}\right|$ (PREC = Normal) and real‐space projectors were used (LREAL = AUTO). For density functionals of the M06‐family, convergence problems with respect to grids and basis sets are well known. We could not obtain reasonable results with standard settings and PAW potentials for M06 and revM06. For revM06 and M06‐SX, satisfactory results were obtained using the newest GW‐PAW potentials (version 64, H:’_GW’, C:’_GW_new’, O:’_GW_new’, Si:’_GW’, Al:’_GW’), a cutoff of 500 eV, a plane wave basis set for the electronic density, which uses reciprocal lattice vectors with a norm up to 2 times larger than for the wave function, $\left|G_{\text{cut}}\right|$ (PREC = Accurate) and no real‐space projectors were used (LREAL = False). We have used the setting ‘LTBOUNDLIBXC = True’, which enforces that the kinetic energy density is larger than the von Weizsäcker kinetic energy density by using the maximum of both. Furthermore, for the periodic global hybrid calculations, gas phase molecules were computed in the same unit cell as the zeolite.

*ω*B97 is the only one of the range‐separated functionals that contains no global HF exchange and instead switches from 0% to 100%, according to the range‐separation parameter, which can be activated in VASP (LRHFCALC = True). All other range‐separated *ω*B97‐functionals include global HF exchange, which is not implemented in the latest VASP version (6.4.3). We have added the combination of global and long‐range range‐separated HF exchange, both for the calculations with plane waves as well as the PAW‐corrections to a local VASP version 6.4.3. The convergence correction used to treat the integrable singularity of the Coulomb potential in reciprocal space within HF exchange was not modified and is thus the same as for only range‐separated HF exchange. We did not find this to be an issue for the cases considered here, which use for any computed energy difference the same (very large) cells for all species and which are generally well converged with respect to k‐point sampling when using only the Γ‐point.

All DLPNO‐CCSD(T) (‘TightPNO’ setting, exact Coulomb and HF exchange) and RI‐MP2 ((RIJCOSX^[^ ^45^ ^]^ with grid X6) calculations were performed with ORCA in version 4.2.1.^[^ ^46^ ^]^ We use the original DLPNO‐CCSD(T)‐version, later termed DLPNO‐CCSD(T0). Following suggestions in the literature,^[^ ^47^ ^]^ we compare here briefly with a subsequently introduced improvement termed DLPNO‐CCSD(T1).^[^ ^48^ ^]^ As shown in Figure S13 in the Supporting Information for the cc‐pVDZ basis set, the agreement is good, with a maximum deviation of 2 kJ mol^−1^ and a mean absolute deviation of about 1 kJ mol^−1^.

The Dunning basis sets cc‐pVXZ^[^ ^49^, ^50^ ^]^ (X = 2, 3, 4) were used with corresponding auxiliary basis sets for RIJ and RI‐MP2.^[^ ^51^ ^]^

RI‐RPA calculations were performed with Turbomole version 7.4.1. DFT calculations with def2‐TZVPP and def2‐QZVPP^[^ ^52^, ^53^ ^]^ basis sets were performed with Turbomole,^[^ ^54^ ^]^ mainly with version 7.7.1, which is linked against libxc, version 5.2.3. All Turbomole calculations use the resolution of the identity (RI) approximation for the Coulomb part with appropriate basis sets,^[^ ^55^ ^]^ while HF exchange is computed exactly.

D4‐dispersion contributions were computed using the stand‐alone program in version 3.4.0. For the r^2^SCAN‐functionals, the specific TZ‐parametrization was employed for D4‐calculations with the def2‐TZVPP basis set.^[^ ^56^ ^]^ The VV10^[^ ^57^ ^]^ calculations were performed nonself‐consistently (post‐SCF), which has been proven to lead only to a negligible loss of accuracy.^[^ ^58^ ^]^ Periodic computations on cluster models use orthorhombic cells with cell parameters 36.0, 32.5, 33.9 Å while a reduced cell‐size was used for hybrid functionals: 30.0, 26.0, 27.0 Å.

The Cartesian coordinates of all periodic and nonperiodic structures are provided in the SI as xyz‐ and Poscar‐files. All total energies are provided as a csv‐file. Additionally, all reaction energies and errors discussed in this work are provided also as a csv‐file along with a Python‐script that generates this data from the total energies.

## 3 Results and Discussion

### 3.1 Test Set

**Figure 1:**
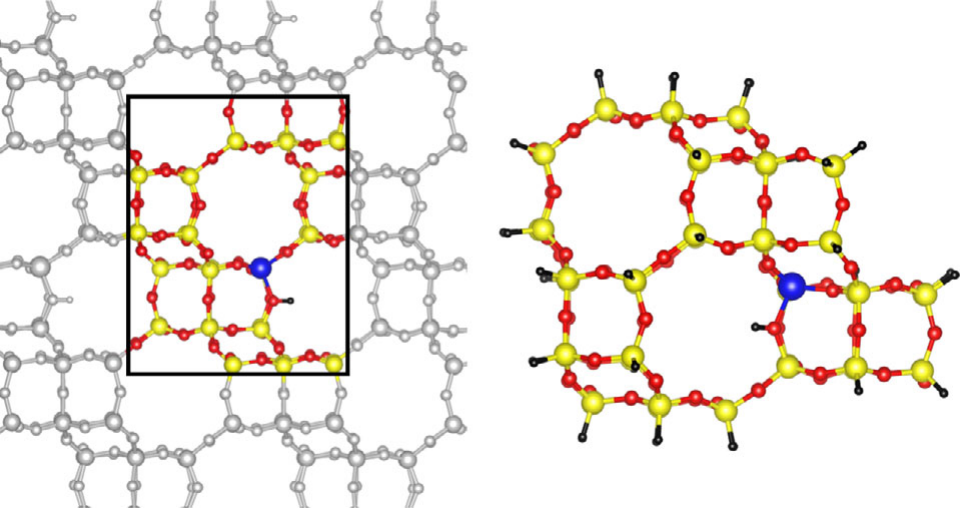
Left: Periodic structure of the CHA zeolite. The unit cell is indicated. Right: Cluster model of the CHA zeolite with 46 T‐sites. Color code: Yellow: Si, blue: Al, red: O, and black: H.

All reactions were studied for the H‐SSZ‐13 zeolite in the chabazite (CHA) structure and employ the unit cell with 36T‐atoms, of which one was substituted with Al (Si/Al = 35). The unit cell is shown in **Figure** **1**, where the lattice constants were optimized at the PBE‐D3 level of theory in previous work (*a* = *b* = 13.625 Å and *c* = 15.067 Å).^[^ ^59^ ^]^ All structures and transition states were then fully optimized (PBE‐D3) with respect to the atomic positions while keeping the lattice constants fixed. The employed cluster model with 46T‐sites was cut from the bulk structures and is also identical to that used by us in previous studies. Dangling Si—O bonds were replaced by Si—H bonds pointing in the same direction with a fixed Si—H bond distance 1.489 Å.

The clusters, cut from PBE‐D3‐optimized periodic models, are not reoptimized and all energies are thus compared using single‐point energy calculations. Such an approach was also found to be appropriate by Rybicki et al. when compared to reoptimization with MP2.^[^ ^18^ ^]^ Nevertheless, we performed a few test calculations to judge the effect of the structure on the barrier. Four transition states of our test set, including the corresponding reference states, were reoptimized at the HSE06‐D3^[^ ^60^ ^]^ level of theory using periodic calculations. We then compare these HSE06‐D3 results with those obtained as single points on the PBE‐D3 structures (HSE06‐D3//PBE‐D3). The result is encouraging, with all deviations below 2 kJ mol^−1^, see Table S4 in the Supporting Information. Relaxed potential energy scans of the corresponding reactions are shown in Figure S9–S12 in the Supporting Information. It can be seen that the curves for HSE06‐D3 and HSE06‐D3//PBE‐D3 overlap closely, thus corroborating the approach to perform hybrid DFT calculations as a single point calculation on top of a structural optimization with PBE‐D3, at least for the tested cases.

**Scheme 1:**
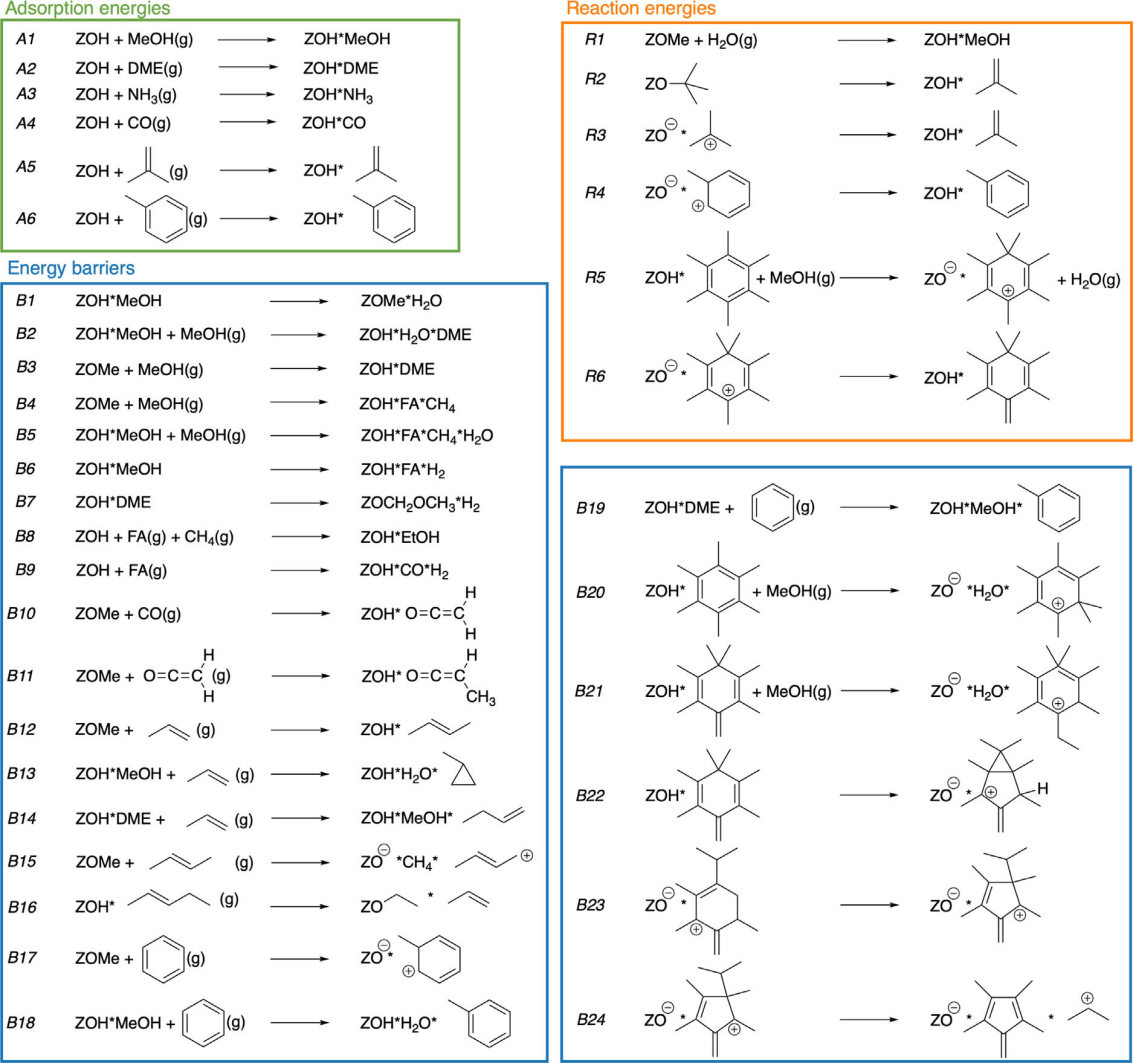
Investigated adsorptions, reactions and reaction barriers.

To study the accuracy of density functionals for reactions in zeolites, we have compiled a test set of 24 barriers, six adsorption reactions, and six reactions of adsorbed species (see **Scheme** **1**). This test set is aimed at the reactivity of the MTO process, and all of these examples were taken from previous work, but all relevant data (Cartesian coordinates and total energies) are provided here as supporting information. The adsorption reactions (A1–A6) include common species such as MeOH, DME, and hydrocarbons such as isobutene and toluene. Reactions (R1–R6) include the protonation of isobutene and toluene to the corresponding cations, as well as the formation of an SMS and the methylation of hexamethylbenzene.

Many zeolite‐catalyzed reactions, such as the MTO process, take place at high temperatures (for example, 400 °C). Reactants and products are often gas phase species and adsorption is in many cases unfavorable at these high temperatures, that is, the Gibbs free energy of adsorption is positive. This has the important consequence that for a reaction to take place, a molecule has to first adsorb, which is unfavorable, and has to then overcome the intrinsic barrier. The apparent barrier is then the activation barrier as referenced to the reactant in the gas phase rather than the adsorbed reactant. When two reactants react with each other, there are of course more options, as both can be either adsorbed or not in the reference state to which we measure the apparent barrier. We note in passing that most constrained MD approaches, such as umbrella‐ or blue‐moon sampling, do not allow to compute adsorption free energies and it is therefore common practice to start with all reactants coadsorbed. Most barriers computed with MD are therefore by construction intrinsic barriers, which may or may not coincide with the apparent barriers depending on the specific case. We also note that adsorption free energies computed using the harmonic oscillator approximation are generally expected to be too weak, as the entropy of (especially weakly bound) adsorbates is underestimated. For harmonic apparent activation free energies this is expected to give a partial error cancellation with the electronic activation energy that is typically too low for the often applied nonhybrid functionals, in particular the default PBE‐D2 or PBE‐D3 case.

When a barrier is referenced to a reactant in the gas phase, this means that the error is the sum of the error of the adsorption step and of the error of the intrinsic barrier. In practice only the overall error matters, while for analysis it is interesting to separate out the adsorption error by using an adsorbed reference state. We have decided to use a reference state where MeOH and DME are adsorbed, since these molecules adsorb relatively strongly and have a well‐defined structure. Furthermore, for reactions involving methylated benzenes, we also use adsorbed reference states, also because these species are effectively trapped in the zeolite. For initial coadsorbed states which are weakly bound, such as CO and an SMS (ZOMe) as in B10, we do not use an adsorbed reference because it is not very stable and because the structures are not well‐defined, as the adsorbate is only loosely bound.

The investigated barriers involve standard methylation reactions of methanol to DME and stepwise and concerted methylation of propylene and benzene. These are among the most commonly studied reactions in zeolite catalysis and are relevant for the dehydration of methanol to DME and also for the MTO process. Furthermore, reactions relevant for initiation, such as dehydrogenation or methylation of CO and ketene, are also studied. Lastly, reactions from the aromatic cycle (paring and side‐chain mechanism) are also included.^[^ ^59^, ^61^, ^62^ ^]^

### 3.2 Periodic Versus Nonperiodic Calculations

In many cases, it is not possible to perform the calculation of a high‐level method for periodic boundary conditions (PBC), for example, for DLPNO‐CCSD(T). We therefore resort to calculating a correction term (high level minus low level) evaluated on the cluster model and add it to the low‐level result obtained for the periodic model

(1) $$E_{\text{high}\;\text{level}}^{\text{approx}\text{.}\;\text{PBC}}=E_{\text{low}\;\text{level}}^{\text{PBC}}+\left(E_{\text{high}\;\text{level}}^{\text{cluster}}-E_{\text{low}\;\text{level}}^{\text{cluster}}\right)$$

**Figure 2:**
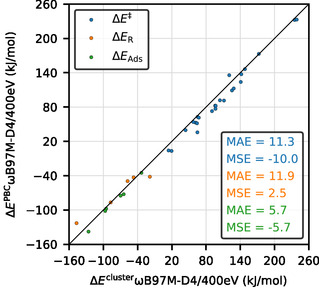
Parity plot for the reaction energies, adsorption energies, and energy barriers comparing periodic *ω*B97M‐D4 calculations for both periodic and cluster‐models.

This approach can therefore be understood as adding a cluster‐based correction to the low‐level result obtained for the PBC model. Alternatively, writing the same equation as

(2) $$E_{\text{high}\;\text{level}}^{\text{approx}\text{.}\;\text{PBC}}=E_{\text{high}\;\text{level}}^{\text{cluster}}+\left(E_{\text{low}\;\text{level}}^{\text{PBC}}-E_{\text{low}\;\text{level}}^{\text{cluster}}\right)$$

the approach can also be understood as adding a low‐level‐based correction for the difference between PBC and cluster model to the high‐level result obtained for the cluster model. With this approach, we therefore rely on the low‐level method adequately describing the difference between cluster and PBC models similar to how the high‐level method would. It is for most ab initio methods not clear, what the actual result of the high‐level calculation evaluated on a periodic system would be. For many of the studied hybrid functionals, the periodic calculation is possible, and this allows us to study the validity of adding a correction term to a PBE‐D3 calculation. **Figure** **2** shows all energies obtained for the periodic systems as a function of those for the cluster model for *ω*B97M‐D4. The obtained energies agree qualitatively, but there are significant differences with mean absolute deviations on the order of 10 kJ mol^−1^.

**Figure 3:**
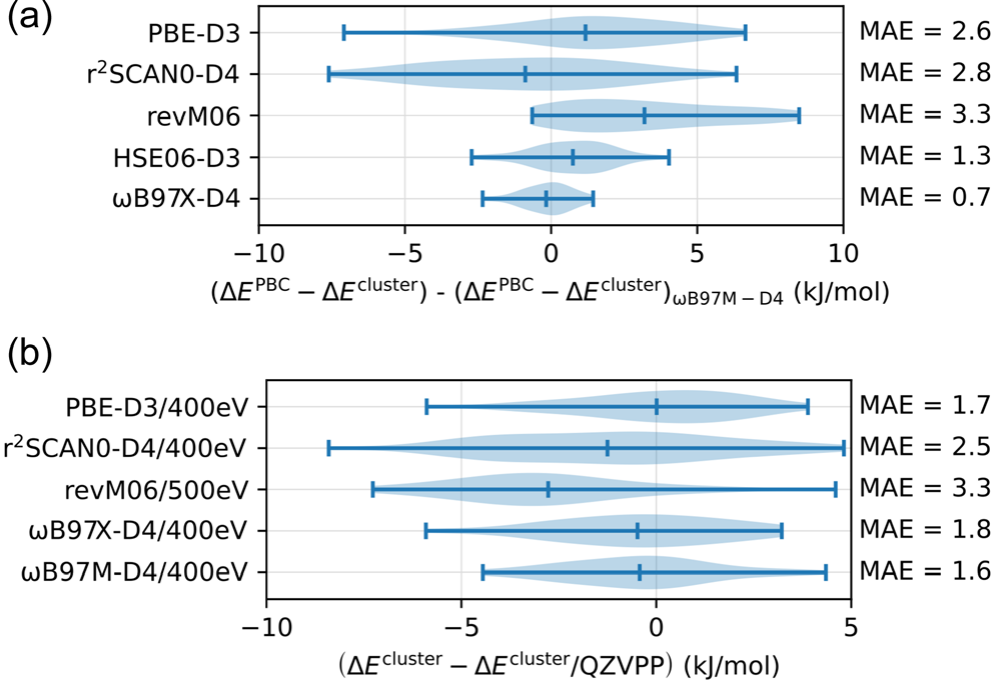
Violin plots for a) the difference between periodic calculations for the CHA‐crystal and for the cluster models. The difference is given for various methods relative to the same quantity, as computed with *ω*B97M‐D4. b) The difference between plane‐wave calculations on cluster models and GTO‐calculations (QZVPP) on the same cluster models. For (a) and (b), all differences between reaction energies, adsorption energies, and energy barriers are considered.

**Figure** **3**a shows how the difference between PBC and cluster model for reaction energies and barriers differs when evaluated with different methods. This is compared by analyzing the variation of the term $\left(E^{\text{PBC}}-E^{\text{cluster}}\right)$ with respect to its value determined with *ω*B97M‐D4. It can be seen that the maximum variation is always below 10 kJ mol^−1^ and in most cases below 5 kJ mol^−1^. Mean absolute deviations are even smaller.

### 3.3 Plane Wave Basis Versus Gaussian Type Orbitals (GTOs)

For the validation of plane‐wave calculations with functionals such as *ω*B97M‐D4 that were rarely or never used with plane‐wave calculations, it is helpful to compare to results obtained with GTOs. To do this, plane‐wave calculations were performed on the cluster models, although it is of course, much more efficient and accurate to directly compute the periodic system. As can be seen in Figure 3b, the agreement of plane‐wave calculations with calculations with the def‐QZVPP‐basis (abbreviated as QZVPP) set is very good for most functionals. In particular for *ω*B97M‐D4, the maximum error is below 5 kJ mol^−1^, and the average error is only 1.6 kJ mol^−1^.

### 3.4 Reference Calculations

The reference method is similar to our initial benchmark article from 2019:^[^ ^26^ ^]^ We use complete basis set (CBS) extrapolated MP2^[^ ^63^ ^]^ calculations and add a correction term^[^ ^64^, ^65^ ^]^ based on the difference between DLPNO‐CCSD(T)^[^ ^66^, ^67^, ^68^, ^69^ ^]^ and MP2. Based on our initial study,^[^ ^26^ ^]^ we have used a computationally less demanding protocol in subsequent studies to save resources. We now briefly explain the reference method and compare it to our usual approach below. Using the exponential three‐point extrapolation procedure,^[^ ^70^ ^]^ HF/CBS(234) results are obtained based on calculations with the cc‐pVXZ basis sets (X = D, T, Q). Compared to most previous work, we computed MP2 with higher precision here, using only the resolution of the identity approximation (RI‐MP2), rather than DLPNO‐MP2. For correlation energies, within the two‐point $l^{-3}$‐extrapolation‐scheme,^[^ ^71^ ^]^ we use MP2/CBS(34) with X = T, Q here, rather than MP2/CBS(23) with X = D, T. Additionally, the difference between DLPNO‐CCSD(T) and MP2 is evaluated using the cc‐pVTZ basis set rather than cc‐pVDZ. While we use the more accurate data here as the reference, our usual approach differs only slightly, giving a mean absolute error (MAE) of 4.0, 3.5, and 2.4 kJ mol^−1^ for adsorption energies, reaction energies, and barriers, which we still consider sufficient in most scenarios, especially given the much higher efficiency. The reference calculations can thus be summarized as:

(3) $$E_{\text{ref}}^{\text{cluster}}=E_{\text{MP2}}^{\text{cor}}/\text{CBS}(34)+E_{\text{HF}}/\text{CBS}(234)+\left(E_{\text{DLPNO}-\text{CCSD(T)}}^{\text{cor}}-E_{\text{MP2}}^{\text{cor}}\right)/\text{pVTZ}$$

(4) $$E_{\text{ref}}^{\text{PBC}}=E_{\text{ref}}^{\text{cluster}}+\left(E_{\omega \text{B97M}-\text{D4}}^{\text{PBC}}-E_{\omega \text{B97M}-\text{D4}}^{\text{cluster}}\right)/400\;\text{eV}$$

As an additional, relevant check to our reference method, we have recomputed adsorption energies on small 4T‐zeolite clusters from a recent benchmark study.^[^ ^31^ ^]^ For seven adsorbates (CH_4_, C_2_H_6_, C_2_H_4_, C_2_H_2_, C_3_H_8_, CO_2_, and H_2_O) both MP2‐F12/cc‐pVTZ‐F12 and PNO‐CCSD(T0)(F12*)/cc‐pVTZ energies were computed therein, to which we can compare the energies obtained with Equation (3). For MP2/CBS, our results differ by a MAE of 0.7 kJ mol^−1^, and for CCSD(T) (i.e., using Equation (3)), by 0.9 kJ mol^−1^. Overall, we thus find good agreement, corroborating the choice of reference method.

### 3.5 Results

**Figure 4:**
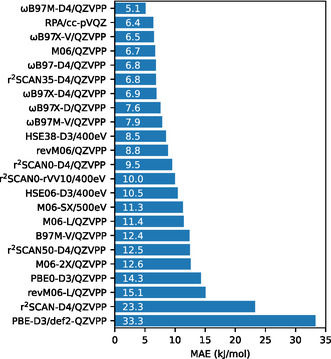
MAE including all types of reactions, adsorption and reaction energies, and transition states. For each functional, only the calculation performed with the best basis set is shown.

Several DFT methods were studied, both using cluster models and periodic models of the CHA crystal. Calculations with GTOs were performed only on the cluster models. Calculations with plane waves were performed both for cluster and periodic models. Not all functionals could be tested in periodic calculations due to limitations of the available PAW potentials for the Minnesota functionals. All DFT calculations are compared with the reference calculations (DLPNO‐CCSD(T) and CBS‐extrapolated MP2, see Section 3.4). For DFT calculations on cluster models, this means that the error is directly determined only by comparing to the reference method as evaluated on the cluster models. For comparison with periodic calculations, this requires to add a correction for the difference between cluster and periodic models, which is based on *ω*B97M‐D4 and adds a small uncertainty, see Section 3.2. The best agreement with reference calculations is of course, expected for the DFT calculations on cluster models, and this is therefore the preferred way to judge the intrinsic accuracy of a DFT‐based method. In this sense, **Figure** **4** is the most compact representation of the results, showing for each functional only one calculation, where possible for a cluster model and with the largest basis set, def2‐QZVPP, abbreviated as QZVPP. Figure 4 shows for each function only the MAE, averaged over all three types of reactions (adsorptions, reactions, and barriers).

**Figure 5:**
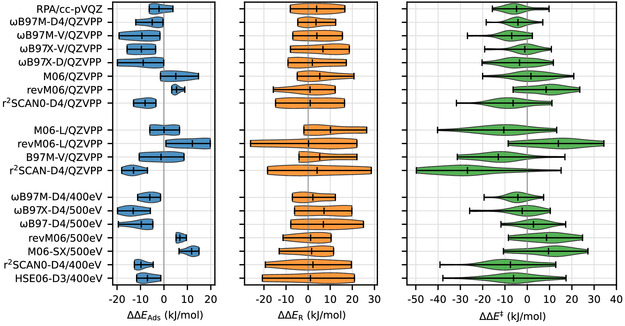
Violin plots showing error distributions separately for reaction energies, adsorption energies, and energy barriers. The upper group shows well‐performing methods for cluster models, the center part only nonhybrid functionals and the lower part shows only results obtained using periodic calculatoins for bulk CHA.

**Figure** **5** shows the distribution of the errors using a violin plot for selected functionals, which perform well in the following groups: 1) Hybrid and post‐KS 2) MGGA, and 3) DFT calculations which we could perform for the periodic models. **Table** **1** lists the MAE and the mean signed error (MSE) for the three types of test reactions (adsorptions, reactions, and barriers) for all calculations. For some functionals, such as r^2^SCAN0‐D4, multiple calculations were performed, both periodic and for clusters, and in the latter case with two GTO basis sets (TZVPP and QZVPP) and plane waves. Results with the TZVPP basis set are given in Table S2 in the Supporting Information.

**Table 1:** MAEs and MSEs in kJ mol^−1^ given separately for adsorption, reactions and barriers.

Method	Basis	Disp.	Class	Model	Δ*E*_Ads_		Δ*E*_R_		$\Delta E^{‡}$	
					MAE	MSE	MAE	MSE	MAE	MSE
RPA	cc‐pVQZ	–	post‐KS	cluster	3.9	−2.1	7.6	3.8	6.7	−4.8
RPA	cc‐pVTZ	–	post‐KS	cluster	11.5	−10.9	10.3	2.5	10.7	−8.3
MP2	CBS(34)	–	post‐HF	cluster	7.9	−7.9	7.1	−3.7	7.6	−5.6
M06	QZVPP	–	MGGA‐hyb.	cluster	5.9	5.1	7.4	5.3	6.7	1.7
revM06	QZVPP	–	MGGA‐hyb.	cluster	5.4	5.4	7.3	0.9	10.1	8.5
revM06	500 eV	–	MGGA‐hyb.	cluster	2.7	2.7	6.5	0.6	7.6	5.0
revM06	500 eV	–	MGGA‐hyb.	PBC	6.8	6.8	6.6	1.1	10.6	8.7
M06‐2X	QZVPP	–	MGGA‐hyb.	cluster	4.6	3.6	10.9	1.2	15.0	12.4
M06‐SX	500 eV	–	MGGA‐hyb.	PBC	11.8	11.8	8.1	1.6	12.0	9.5
*ω*B97M‐V	QZVPP	VV10	MGGA‐hyb.	cluster	9.4	−9.4	8.1	4.0	7.4	−7.0
*ω*B97M‐rV(6.2)	400 eV	rVV10	MGGA‐hyb.	cluster	15.5	−15.5	8.6	1.7	11.4	−10.8
*ω*B97M‐rV(7.1)	400 eV	rVV10	MGGA‐hyb.	cluster	8.0	−8.0	7.9	2.8	6.0	−5.1
*ω*B97M‐rV(8.5)	400 eV	rVV10	MGGA‐hyb.	cluster	1.7	0.6	7.8	4.0	3.3	1.4
*ω*B97M‐D3(BJ)	400 eV	D3	MGGA‐hyb.	PBC	9.3	−9.3	6.1	1.1	7.8	−7.0
*ω*B97M‐D4	QZVPP	D4	MGGA‐hyb.	cluster	5.0	−5.0	5.2	3.5	5.1	−4.2
*ω*B97M‐D4	400 eV	D4	MGGA‐hyb.	cluster	6.0	−6.0	6.2	2.2	5.4	−4.2
*ω*B97M‐D4	400 eV	D4	MGGA‐hyb.	PBC	6.0	−6.0	6.2	2.2	5.4	−4.2
r^2^SCAN0‐D4	QZVPP	D4	MGGA‐hyb.	cluster	8.0	−8.0	10.9	1.0	9.5	−6.3
r^2^SCAN0‐D4	400 eV	D4	MGGA‐hyb.	cluster	10.2	−10.2	11.3	1.4	11.1	−7.8
r^2^SCAN0‐D4	400 eV	D4	MGGA‐hyb.	PBC	9.8	−9.8	10.3	2.2	11.0	−7.5
r^2^SCAN0‐rVV10	400 eV	rVV10	MGGA‐hyb.	PBC	8.8	−8.8	11.0	3.0	10.0	−6.1
r^2^SCAN35‐D4	QZVPP	D4	MGGA‐hyb.	cluster	5.2	−5.2	11.5	0.1	6.1	1.5
r^2^SCAN35‐D4	400 eV	D4	MGGA‐hyb.	PBC	7.1	−7.1	10.6	1.7	7.1	0.1
r^2^SCAN50‐D4	QZVPP	D4	MGGA‐hyb.	cluster	2.3	0.1	12.4	−0.8	15.0	13.3
*ω*B97X‐D	QZVPP	D	GGA‐hyb.	cluster	8.9	−8.9	9.1	1.9	6.9	−3.5
*ω*B97X‐V	QZVPP	VV10	GGA‐hyb.	cluster	9.6	−9.6	11.3	6.7	4.5	−1.1
*ω*B97X‐rV	500 eV	rVV10	GGA‐hyb.	PBC	18.6	−18.6	16.1	8.3	8.7	−6.6
*ω*B97X‐D4	QZVPP	D4	GGA‐hyb.	cluster	11.7	−11.7	9.8	7.5	5.0	−1.7
*ω*B97X‐D4	400 eV	D4	GGA‐hyb.	cluster	13.0	−13.0	11.1	7.3	5.9	−2.1
*ω*B97X‐D4	400 eV	D4	GGA‐hyb.	PBC	13.1	−13.1	11.0	7.2	5.8	−2.3
*ω*B97X‐D3(BJ)	400 eV	D3	GGA‐hyb.	PBC	14.7	−14.7	9.7	5.8	7.0	−3.0
*ω*B97‐D4	QZVPP	D4	GGA‐hyb.	cluster	8.7	−8.7	10.1	6.4	5.5	3.0
*ω*B97‐D4	500 eV	D4	GGA‐hyb.	PBC	9.7	−9.7	11.5	6.9	5.8	2.8
HSE06‐D4	400 eV	D4	GGA‐hyb.	cluster	13.7	−13.7	11.3	1.1	14.8	−11.6
HSE06‐D3	400 eV	D3	GGA‐hyb.	cluster	7.5	−7.5	14.9	0.7	10.9	−7.1
HSE06‐D3	400 eV	D3	GGA‐hyb.	PBC	7.0	−7.0	14.1	1.1	10.4	−6.2
HSE38‐D3	400 eV	D3	GGA‐hyb.	cluster	5.4	−5.4	12.7	−0.9	8.5	3.7
HSE38‐D3	400 eV	D3	GGA‐hyb.	PBC	5.1	−5.1	11.8	0.2	8.5	4.0
PBE0‐D3	QZVPP	D3	GGA‐hyb.	cluster	13.7	−13.7	10.7	0.2	15.3	−13.2
PBE0‐D4	QZVPP	D4	GGA‐hyb.	cluster	13.0	−13.0	9.0	1.5	14.8	−12.3
M06‐L	QZVPP		MGGA	cluster	4.9	0.0	11.0	10.1	13.2	−10.5
M06‐L	500 eV		MGGA	PBC	5.7	5.0	13.0	12.2	12.0	−9.8
revM06‐L	QZVPP		MGGA	cluster	12.1	12.1	15.4	0.2	15.7	14.0
revM06‐L	500 eV		MGGA	PBC	13.8	13.8	14.8	0.3	15.9	13.8
B97M‐V	QZVPP	VV10	MGGA	cluster	6.2	−1.2	9.0	5.2	14.8	−13.0
r^2^SCAN‐D4	QZVPP	D4	MGGA	cluster	13.0	−13.0	14.3	4.1	28.1	−26.8
PBE‐D3	QZVPP	D3	GGA	cluster	18.8	−18.8	23.4	3.5	39.4	−38.1
PBE‐D3	400 eV	D3	GGA	cluster	20.3	−20.3	25.2	3.4	39.0	−37.7
PBE	400 eV		GGA	PBC	29.4	29.4	22.3	11.5	18.8	1.8
PBE‐D2	400 eV	D2	GGA	PBC	19.1	−19.1	23.0	5.3	38.3	−37.8
PBE‐D3	400 eV	D3	GGA	PBC	19.8	−19.8	22.7	2.3	36.8	−35.8
PBE‐D3(BJ)	400 eV	D3	GGA	PBC	20.8	−20.8	20.4	2.9	37.5	−36.4
PBE‐D4	400 eV	D4	GGA	PBC	20.3	−20.3	20.1	3.5	36.8	−35.8

Possibly the intrinsically most accurate and computationally most demanding DFT‐method is the RPA. As usual, we perform the RPA‐calculations using a Kohn–Sham wave function based on the PBE‐functional and our best result is RPA/cc‐pVQZ. As with MP2 and CCSD(T), the error of the noncounterpoise‐corrected HF‐energy is unreasonably high with the cc‐pVTZ basis set due to BSSE. Test calculations on 2T‐cluster models show that beyond the cc‐pVQZ basis set, it is mostly the accuracy of the correlation energy that is limited. Unfortunately, in agreement with previous reports, we have found that CBS‐extrapolation does not improve the RPA‐results, in contrast to MP2. For the 2T‐cluster model, RPA/cc‐pVQZ overbinds MeOH (reaction A1) by about 5 kJ mol^−1^ compared to the estimated RPA/CBS‐limit. The MSE error of RPA/cc‐pVQZ with respect to the reference method is −2.1 kJ mol^−1^ for adsorption energies (A1–A6) for 46T‐models, which is indicative of BSSE since RPA is expected to underbind.^[^ ^31^ ^]^ In Figure S1 and Table S3 in the Supporting Information, we additionally analyze the errors of barriers separately for the intrinsic barriers (B1, B6, B7, B22, B23, and B24), for which the MAE of RPA/cc‐pVQZ is only 3.9 kJ mol^−1^. We generally observe an underestimation of barriers, in agreement with results reporte for the BH76 barrier heights test.^[^ ^72^ ^]^ Overall, RPA/cc‐pVQZ performs well, but adsorption energies and apparent activation energies are clearly not fully converged with respect to basis set. We also list RPA/cc‐pVTZ in Table 1, which leads to MAEs on the order of 10 kJ mol^−1^ and is, despite high computational cost, less accurate than many density functionals that can be performed more easily.

The least accurate and computationally least demanding tested method is dispersion‐corrected PBE‐D*n* (*n* = 2, 3, 4), which is probably the most widespread functional in zeolite catalysis. The variation in the MAE between the different dispersion variants (D2, D3(zero damping), D3(BJ) (Becke–Johnson damping), D4) is always below 4 kJ mol^−1^ and mostly even smaller. Dispersion‐corrected PBE is found to overbind on average by about 20 kJ mol^−1^ in adsorption energies, which is in line with reports in the literature,^[^ ^13^, ^17^, ^26^, ^31^ ^]^ as also discussed in the introduction. The well‐known underestimation of barrier heights, combined with overestimated adsorption energies, leads to large errors in barriers, with MAEs of about 35 kJ mol^−1^ for the PBE‐D*n* variants. For illustration, we have included PBE (without dispersion correction) in Table 1, which underbinds on average by 29.4 kJ mol^−1^ in adsorption energies. Through error cancellation between lacking dispersion interaction and barrier underestimation, the MAE for barriers is 18.8 kJ mol^−1^ and the MSE is 1.8 kJ mol^−1^. Overbinding is found for all dispersion‐corrected functionals in our test set, and so is error cancellation between intrinsic barriers and adsorption in the calculation of apparent barriers. The high MAEs for PBE and PBE‐D*n* indicate that it is not a reliable functional for barriers, especially when an adsorption step is involved. However, for some notable cases, the error is not that large, that is, for the intrinsic barrier for SMS‐formation (B1) or for the intrinsic barrier for propene elimination in the aromatic cycle (B24). Interestingly, in a recent MD‐study using RPA‐trained machine learning, PBE‐D2 was found to perform very well for olefin isomerization barriers. As usual in MD‐studies, the results are referenced to adsorbed species, thus giving intrinsic barriers without error in adsorption. Furthermore, the barriers in that work are relatively low (<80 kJ mol^−1^).

We now move from PBE up Jacob's ladder to MGGA functionals. M06‐L^[^ ^73^ ^]^ and B97M‐V^[^ ^74^ ^]^ perform best, but we were able to obtain PW‐DFT results only for M06‐L, where we employed different PAW potentials and more accurate settings (see computational details), as for all Minnesota functionals. M06‐L and B97M‐V both work well for adsorption and reaction energies (MAE < 10 kJ mol^−1^) and reasonably well for barriers, which are usually underestimated, as expected, with MAEs of 13 and 14 kJ mol^−1^. A revised version of M06‐L, revM06‐L,^[^ ^75^ ^]^ performs less well and is notably the only MGGA that predicts on average too high barriers. The r^2^SCAN functional is, from a purist's perspective, the most attractive functional as its design principle is to fulfill conditions that the exact (unknown) exchange‐correlation functional obeys. r^2^SCAN‐D4^[^ ^76^, ^77^ ^]^ performs reasonably well for adsorption and reaction energies but gives (after PBE‐D*n*) the second‐largest errors for barriers of the functionals listed here.

We will now discuss hybrid functionals, both MGGAs and GGAs. The most accurate functionals are the range‐separated *ω*B97‐functionals developed by Head–Gordon and coworkers. There are a few of these functionals, which we will first explain: The most accurate in our tests are the functionals deriving from the VV10‐dispersion‐corrected MGGA‐hybrid *ω*B97M‐V.^[^ ^78^ ^]^ Using the same parametrization for the non‐van‐der‐Waals part, D3‐ and D4‐parametrizations have been proposed, *ω*B97M‐D3(BJ)^[^ ^58^ ^]^ and *ω*B97M‐D4.^[^ ^79^ ^]^ Analogously, for the GGA‐hybrid *ω*B97X‐V,^[^ ^80^ ^]^ D3(BJ)‐ and D4‐parametrizations were proposed, *ω*B97X‐D3(BJ)^[^ ^58^ ^]^ and *ω*B97X‐D4.^[^ ^79^ ^]^ These functionals should not be confused with *ω*B97X^[^ ^81^ ^]^ (no dispersion), *ω*B97X‐D,^[^ ^82^ ^]^ and *ω*B97X‐D3^[^ ^83^ ^]^ (zero damping), which all have a differently parametrized individual non‐vdW part. Additionally, there is the *ω*B97‐functional,^[^ ^81^ ^]^ which is unique since it contains no global HF exchange. This is of interest as it is the only range‐separated functional that can be used in an unmodified VASP version at the time of writing (version 6.4.3 or older).

Notably, *ω*B97M‐D4 is the best‐performing functional, which is better than MP2/CBS(34) and RPA/cc‐pVQZ, and is the only functional with an overall MAE below 6 kJ mol^−1^. Besides a small MAE, the *ω*B97‐functionals also have least outliers, leading to a narrow error distribution, see Figure 5. It is also noteworthy that of all DFT‐methods (except RPA), the *ω*B97‐functionals improve most consistently with basis set size, here when we go from TZVPP to QZVPP. We have not performed QZVPPD calculations as in the original publication, due to the high computational demand and convergence issues. *ω*B97M‐V performs also well but binds stronger than *ω*B97M‐D4, leading to a higher overall error. The D3(BJ) version *ω*B97M‐D3(BJ) is closer to *ω*B97M‐V and thus also performs a bit worse than *ω*B97M‐D4. For *ω*B97X‐V and *ω*B97M‐D4, it is the other way around: The MAE of *ω*B97X‐V/QZVPP is 6.5 kJ mol^−1^, while that of *ω*B97X‐D4 is 6.9 kJ mol^−1^.

The VV10^[^ ^57^ ^]^‐dispersion part required for *ω*B97M‐V is generally not available in plane‐wave codes, but the *ω*B97M‐rV‐alternative^[^ ^84^ ^]^ based on rVV10^[^ ^84^ ^]^ has been developed, which requires, however, a refitting of the *b*‐parameter. Like with the parent VV10‐functional, the *c*‐parameter is typically left at 0.0093 and only *b* is fitted, where larger values of *b* lead to stronger binding. Generally, rVV10 binds stronger and therefore the parametrization tied to the rPW86PBE‐functional used a value of *b *= 5.9 for VV10 and *b *= 6.3 for rVV10. Mardirossian and Head‐Gordon proposed a value of *b *= 6.2 for *ω*B97M‐rV, which we term *ω*B97M‐rV(6.2) here. We find that *ω*B97M‐rV(6.2) overbinds severely, even compared to *ω*B97M‐V. Using the common approach of scanning *b* in steps of 0.1, we find that *ω*B97M‐V is best reproduced by *b *= 7.1 and that the overall error of our test set is minimized for *b *= 8.5. We do not advertise the use of a refitted *b*‐parameter, but simply list the results in Table 1. There are very few studies on *ω*B97M‐rV, but based on the available data in the original publication,^[^ ^84^ ^]^ one would expect it to behave similarly to *ω*B97M‐V. The reason for this unexpected deviation is unclear and difficult to investigate since rVV10 is rarely used in GTO‐codes and is, to our knowledge only implemented in Q‐Chem. We did not find the rVV10‐contribution to depend significantly on the plane‐wave cutoff or on the type of PAW potential used (regular, hard, GW, and semicore‐valence).

M06^[^ ^85^ ^]^ and its revised version, revM06^[^ ^86^ ^]^ also perform well, with overall MAEs of 6.7 and 8.8 kJ mol^−1^, respectively. As mentioned above, we used more accurate PAW potentials and as‐accurate‐as‐possible settings for the Minnesota functionals. Despite this effort, plane‐wave results for M06 were still relatively far off from GTO‐results (QZVPP), with deviations sometimes up to 15 kJ mol^−1^ and these results are therefore omitted. These problems with M06 can be attributed due its overparametrization, which made it vulnerable to all kinds of numerical issues, for example with respect to integration grids, basis sets, and here PAW potentials. revM06 was reparametrized aiming for a smoother parametrization, and we indeed find better convergence and decent agreement between plane wave and GTO calculations. M06‐SX^[^ ^87^ ^]^ a short range HF‐variant, and M06‐2X^[^ ^85^ ^]^ lead to overall MAEs of 11.3 and 12.6 kJ mol^−1^ and thus perform worse than M06 and revM06. All of these functionals include no dispersion correction and systematically underbind in adsorption energies and overestimate barriers. Nevertheless, it is noteworthy that this behavior is very systematic. For the Minnesota functionals, increasing the basis set from TZVPP to QZVPP reduces BSSE, therefore also error cancellation in adsorption energies and in the end thus worsens the results for adsorption. Apart from that the basis set effect is relatively small and unsystematic.

The r^2^SCAN0‐D4^[^ ^56^ ^]^ functional with 25% HF exchange works similarly well, also when it is applied with the rVV10^[^ ^56^ ^]^ correction instead of D4. We have also tested the proposed r^2^SCAN50‐D4^[^ ^56^ ^]^ functional with 50% HF exchange, which clearly over‐corrects the somewhat too low barriers observed for r^2^SCAN0‐D4. As an intermediate case, we have tested r^2^SCAN35‐D4 with 35% HF exchange, which has the desired effect of shifting barriers up and lowering the MAE from 11.8 to 6.7 kJ mol^−1^. However, this is clearly achieved with error compensation. Intrinsic barriers are overestimated and compensate for too strong adsorption. This is similar for HSE06‐D3,^[^ ^60^ ^]^ which is commonly used for plane‐wave calculations with 25% HF exchange and the rarely used modification HSE38‐D3 with 38% HF exchange,^[^ ^88^ ^]^ which performs better but relies on error cancellation. Another interesting point is the large difference between PBE0‐D3 and HSE06‐D3, where HSE06‐D3 binds weaker and thus performs better. This is due to the D3‐parametrization in version 3.1 of the dftd3 program, which gives untypically weak D3‐binding energies. If one applies the D3‐parameters of PBE0, HSE06 and PBE0 are very similar and this is also what we find for the latest D4‐parametrization (PBE0‐D4 and HSE06‐D4).

Besides average errors, another important criterion is the occurrence of significant outliers. This can be studied for selected cases in the violin plots (Figure 5), which illustrate the maximum, minimum, and mean deviation, as well as the overall shape of the distribution. At the top, Figure 5 shows the best performing DFT‐methods for cluster models, where RPA/cc‐PVQZ has not only the 2nd‐smallest absolute error but also a narrow distribution and small maximum/minimum deviations. Despite its good performance in terms of the MAE, M06 has a broader error distribution for barriers (±20 kJ mol^−1^), while r^2^SCAN0‐D4 has the broadest error distribution for barriers of the depicted MGGA‐hybrid functionals. Figure 5 also shows the performance of the studied MGGAs for cluster models, where one can also see relatively broad error distributions for barriers predicted by M06‐L and r^2^SCAN‐D4. Lastly, Figure 5 shows the performance of functionals, which could be computed using periodic models of the zeolite crystal. Not surprisingly, the range‐separated *ω*B97‐functionals perform best. As noted above, to compare the periodic calculations with the cluster models, we rely on computing the difference between PBC and cluster models with *ω*B97M‐D4.

## 4 Conclusion

We have investigated density functional methods for a test set of zeolite reactions involving mainly barriers, but also adsorptions and other chemical reactions. Most calculations—as well as the reference calculations—were performed on large 46T‐zeolite models that are close to the periodic bulk model. The reference calculations are CBS‐extrapolated MP2‐calculations with a CCSD(T)‐MP2 correction based on DLPNO‐CCSD(T)/cc‐pVTZ. The difference between bulk and cluster model is described similarly by different DFT‐methods, corroborating the use of high‐level cluster corrections in combination with periodic calculations.

Nonhybrid functionals, such as PBE, are generally known to underestimate barriers. This is also observed for zeolites and is additionally connected to an overstabilization of zwitterionic intermediates, where the adsorbate is a carbocation. An additional issue is that adsorption energies contain a large vdW‐contribution that can only be addressed using dispersion‐corrected functionals. However, dispersion‐corrected functionals usually overbind. To obtain kinetically relevant apparent activation energies in zeolite catalysis at elevated temperatures, barriers often need to be evaluated with respect to reactants in the gas phase. Such an apparent activation barrier will therefore contain an adsorption contribution and its error. Unfortunately, for most density functionals, too strong adsorption and too low intrinsic barriers add up to an even larger underestimation of apparent activation barriers. All nonhybrid functionals, such as PBE‐D3 or M06‐L may perform well for selected reactions but lead in general to large uncertainties especially in reaction barriers.

The Minnesota hybrid functionals, M06 and its revised versions revM06 perform overall well. Applied in the originally parametrized way, that is, without additional dispersion corrections, these functionals tend to underbind in adsorption reactions and to overestimate apparent barriers. The original M06‐functionals are challenging for PAW potentials, and we were not able to reproduce GTO results satisfactorily. This works better for the revised versions, however, not with the standard PAW potentials.

The range‐separated hybrid *ω*B97‐functionals work well for reactions catalyzed by acidic zeolites. In particular, *ω*B97M‐D4 performs very well, with an overall MAE of only 5.1 kJ mol^−1^. Plane‐wave calculations were shown to reliably reproduce the results of GTO calculations for the *ω*B97‐functionals. Importantly, these functionals can be directly applied to periodic zeolite models using plane‐wave DFT. Compared to ab initio calculations on cluster models or RPA calculations on periodic models, periodic hybrid density functional calculations have a very low computational demand, both in terms of memory and CPU time. Additionally, post‐HF and post‐KS methods generally suffer from slow basis set convergence. We therefore believe that the accuracy and efficiency of the *ω*B97‐functionals, in particular *ω*B97M‐D4, make them a very good choice for periodic DFT calculations on acidic zeolites.

In this work, we have investigated the accuracy of electronic structure methods, evaluated at stationary points of the potential energy surface. The most straightforward application of accurate hybrid density functional calculations is within the harmonic approximation as a single‐point calculation on top of the optimization with a more efficient, but less accurate functional such as PBE‐D3. While this is expected to correct a large part of the error of the electronic structure method, it does, of course, not address the limitation of the harmonic approximation. The main approach in zeolite catalysis to go beyond the harmonic approximation is via constrained MD simulations. Due to the large number of required MD‐steps, the electronic structure method is typically limited to GGA‐DFT, mostly dispersion‐corrected PBE. More recently, machine‐learning based on more accurate methods (RPA) has been used as promising approach to perform MD simulations at a higher level of theory.^[^ ^32^, ^33^, ^34^, ^35^ ^]^ Also in this respect, periodic hybrid density functionals could be useful alternatives to the RPA, since they will allow much more rapid generation of training data.

## Conflict of Interest

The authors declare no conflict of interest.

## Supporting information

Supplementary Material

## Data Availability

The data that support the findings of this study are available in the supplementary material of this article.
