# Supplementary material for: Accurate and Efficient Description of Acidic Zeolites with Plane‐Wave Density Functional Theory Using Range‐Separated Hybrid Functionals
Source: Chemphyschem. 2025 Jun 30;26(15):e202500147. doi: 10.1002/cphc.202500147 (PMC12321285; doi:10.1002/cphc.202500147)
Supplement: Supplementary file 1 — Supplementary Material [file CPHC-26-e202500147-s001.zip › cphc202500147-sup-0001-SuppData-S1.pdf]

Supporting Information: Accurate and Efficient Description of Acidic Zeolites with plane-wave DFT using Range-Separated Hybrid Functionals

Philipp Huber,[a] Philipp N. Plessow[a,*]

Contents

S1. Details of Program packages, versions and settings S2

S2. Results with TZVPP basis S3

S3. Detailed comparison of some methods for all test reactions S4

S4. Analysis of intrinsic barriers S6

S5. Analysis of basis set convergence for 2T cluster models S7

S6. Effect of geometry optimization using a hybrid functional S10

S7. DLPNO-CCSD(T1) vs. DLPNO-CCSD(T0) S12

S1. Details of Program packages, versions and settings

Table S1. A

method	program	non-default settings
RPA/cc-pVQZ	Turbomole 7.4.1	
RPA/cc-pVTZ	Turbomole 7.4.1	
M06/TZVPP	Turbomole 7.7.1	grid m5
M06/QZVPP	Turbomole 7.7.1	grid 5
revM06/TZVPP	Turbomole 7.7.1	grid 5
revM06/QZVPP	Turbomole 7.7.1	grid 5
revM06/500eV[b]	Vasp 6.4.1	PREC=Accurate, LTBOUNDLIBXC=.TRUE., GW-PAWs
revM06/500eV	Vasp 6.4.1	PREC=Accurate, LTBOUNDLIBXC=.TRUE., GW-PAWs
M06-2X/TZVPP	Turbomole 7.7.1	grid m5
M06-2X/QZVPP	Turbomole 7.7.1	grid m5
M06-SX/500eV	Vasp 6.4.1	PREC=Accurate, LTBOUNDLIBXC=.TRUE., GW-PAWs
ωB97M-V/TZVPP	Turbomole 7.7.1	grid 5
ωB97M-V/QZVPP	Turbomole 7.7.1	grid 5
ωB97M-rV(6.2)/400eV[b]	Vasp 6.4.3 (modified)	PREC=Accurate, LTBOUNDLIBXC=.TRUE.
ωB97M-rV(7.1)/400eV[b]	Vasp 6.4.3 (modified)	PREC=Accurate, LTBOUNDLIBXC=.TRUE.
ωB97M-rV(8.5)/400eV[b]	Vasp 6.4.3 (modified)	PREC=Accurate, LTBOUNDLIBXC=.TRUE.
ωB97M-D3(BJ)/400eV	Vasp 6.4.3 (modified)	PREC=Accurate, LTBOUNDLIBXC=.TRUE.
ωB97M-D4/TZVPP	Turbomole 7.7.1	grid 5
ωB97M-D4/QZVPP	Turbomole 7.7.1	grid 5
ωB97M-D4/400eV[b]	Vasp 6.4.3 (modified)	PREC=Accurate, LTBOUNDLIBXC=.TRUE.
ωB97M-D4/400eV	Vasp 6.4.3 (modified)	PREC=Accurate, LTBOUNDLIBXC=.TRUE.
r^2SCAN0-D4/TZVPP	Turbomole 7.7.1	grid 5
r^2SCAN0-D4/QZVPP	Turbomole 7.7.1	grid 5
r^2SCAN0-D4/400eV[a]	Vasp 6.4.1	PREC=Normal
r^2SCAN0-D4/400eV	Vasp 6.4.1	PREC=Normal
r^2SCAN0-rVV10/400eV	Vasp 6.4.1	PREC=Normal
r^2SCAN35-D4/TZVPP	Turbomole 7.7.1	grid 5
r^2SCAN35-D4/QZVPP	Turbomole 7.7.1	grid 5
r^2SCAN35-D4/400eV	Vasp 6.4.1	PREC=Normal
r^2SCAN50-D4/TZVPP	Turbomole 7.7.1	grid 5
r^2SCAN50-D4/QZVPP	Turbomole 7.7.1	grid 5
ωB97X-D/TZVPP	Turbomole 7.7.1	grid m5
ωB97X-D/QZVPP	Turbomole 7.7.1	grid m5
ωB97X-V/TZVPP	Turbomole 7.7.1	grid 5
ωB97X-V/QZVPP	Turbomole 7.7.1	grid 5
ωB97X-rV/500eV	Vasp 6.4.3 (modified)	PREC=Accurate, GW-PAWs
ωB97X-D4/TZVPP	Turbomole 7.7.1	grid 5
ωB97X-D4/QZVPP	Turbomole 7.7.1	grid 5
ωB97X-D4/400eV[b]	Vasp 6.4.3 (modified)	PREC=Accurate
ωB97X-D4/400eV	Vasp 6.4.3 (modified)	PREC=Accurate
ωB97X-D3(BJ)/400eV	Vasp 6.4.3 (modified)	PREC=Accurate
ωB97-D4/QZVPP	Turbomole 7.7.1	grid 5
ωB97-D4/500eV	Vasp 6.4.3 (modified)	PREC=Accurate, GW-PAWs

continued

method	program	non-default settings
HSE06-D4/400eV[a]	Vasp 6.3.0	PREC=Normal
HSE06-D3/400eV[a]	Vasp 6.3.0	PREC=Normal
HSE06-D3/400eV	Vasp 6.3.0	PREC=Normal
HSE38-D3/400eV[a]	Vasp 6.3.0	PREC=Normal
HSE38-D3/400eV	Vasp 6.3.0	PREC=Normal
PBE0-D3/TZVPP	Turbomole 7.7.1	grid m5
PBE0-D3/QZVPP	Turbomole 7.7.1	grid m5
PBE0-D4/QZVPP	Turbomole 7.7.1	grid m5
M06-L/QZVPP	Turbomole 7.7.1	grid 5
M06-L/500eV	Vasp 6.4.1	PREC=Accurate, LTBOUNDLIBXC=.TRUE., GW-PAWs
revM06-L/QZVPP	Turbomole 7.7.1	grid 5
revM06-L/500eV	Vasp 6.4.1	PREC=Accurate, LTBOUNDLIBXC=.TRUE., GW-PAWs
B97M-V/QZVPP	Turbomole 7.7.1	grid 5
r^2SCAN-D4/QZVPP	Turbomole 7.7.1	grid 5
PBE-D3/def2-QZVPP	Turbomole 7.7.1	grid m5
PBE-D3/400eV[a]	Vasp 5.4.1	PREC=Normal
PBE/400eV	Vasp 5.4.1	PREC=Normal
PBE-D2/400eV	Vasp 5.4.1	PREC=Normal
PBE-D3/400eV	Vasp 5.4.1	PREC=Normal
PBE-D3(BJ)/400eV	Vasp 5.4.1	PREC=Normal
PBE-D4/400eV	Vasp 5.4.1	PREC=Normal
MP2-cor/pVDZ	Orca 4.2.1	RIJCOSX, GridX6
MP2-cor/pVTZ	Orca 4.2.1	RIJCOSX, GridX6
MP2-cor/pVQZ	Orca 4.2.1	RIJCOSX, GridX6
MP2-cor/CBS(23)	Orca 4.2.1	RIJCOSX, GridX6
MP2-cor/CBS(34)	Orca 4.2.1	RIJCOSX, GridX6
HF/pVDZ	Orca 4.2.1	RIJCOSX, GridX6
HF/pVTZ	Orca 4.2.1	RIJCOSX, GridX6
HF/pVQZ	Orca 4.2.1	RIJCOSX, GridX6
HF/CBS(234)	Orca 4.2.1	RIJCOSX, GridX6
DLPNO-CCSD(T)/pVDZ	Orca 4.2.1	TightPNO
DLPNO-CCSD(T)/pVTZ	Orca 4.2.1	TightPNO

[a] cluster model with larger orthorombic cell (36.0 Å, 32.5 Å, 33.9 Å)
[b] cluster model with smaller orthorombic cell (30.0 Å, 26.0 Å, 27.0 Å)

S2. Results with TZVPP basis

Table S2. MAEs and MSEs in kJ/mol given separately for adsorption, reactions and barriers.

Method	Basis	Disp.	Class	Model	ΔE_{Ads}		ΔE_{R}		$\Delta E^{\ddagger}$	
					MAE	MSE	MAE	MSE	MAE	MSE
M06	TZVPP		MGGA-hyb.	cluster	5.7	1.5	8.3	5.7	6.6	-0.1
revM06	TZVPP		MGGA-hyb.	cluster	1.7	0.5	7.5	0.5	7.8	5.6
M06-2X	TZVPP		MGGA-hyb.	cluster	3.1	-1.7	11.2	0.8	12.8	9.5
ωB97M-V	TZVPP	VV10	MGGA-hyb.	cluster	14.3	-14.3	9.6	3.2	10.4	-9.7
ωB97M-D4	TZVPP	D4	MGGA-hyb.	cluster	9.9	-9.9	6.7	2.8	8.0	-7.0
r^2SCAN0-D4	TZVPP	D4	MGGA-hyb.	cluster	10.3	-10.3	11.3	1.0	10.8	-7.6
r^2SCAN35-D4	TZVPP	D4	MGGA-hyb.	cluster	7.5	-7.5	11.8	0.1	6.3	0.3
r^2SCAN50-D4	TZVPP	D4	MGGA-hyb.	cluster	4.1	-3.6	12.5	-1.0	12.9	11.0
ωB97X-D	TZVPP	D	GGA-hyb.	cluster	13.6	-13.6	10.5	1.6	8.8	-6.3
ωB97X-V	TZVPP	VV10	GGA-hyb.	cluster	14.2	-14.2	12.9	6.1	6.7	-3.8
ωB97X-D4	TZVPP	D4	GGA-hyb.	cluster	16.2	-16.2	11.2	7.0	6.8	-4.4
PBE0-D3	TZVPP	D3	GGA-hyb.	cluster	18.0	-18.0	11.9	-0.3	18.0	-15.8

S3. Detailed comparison of some methods for all test reactions

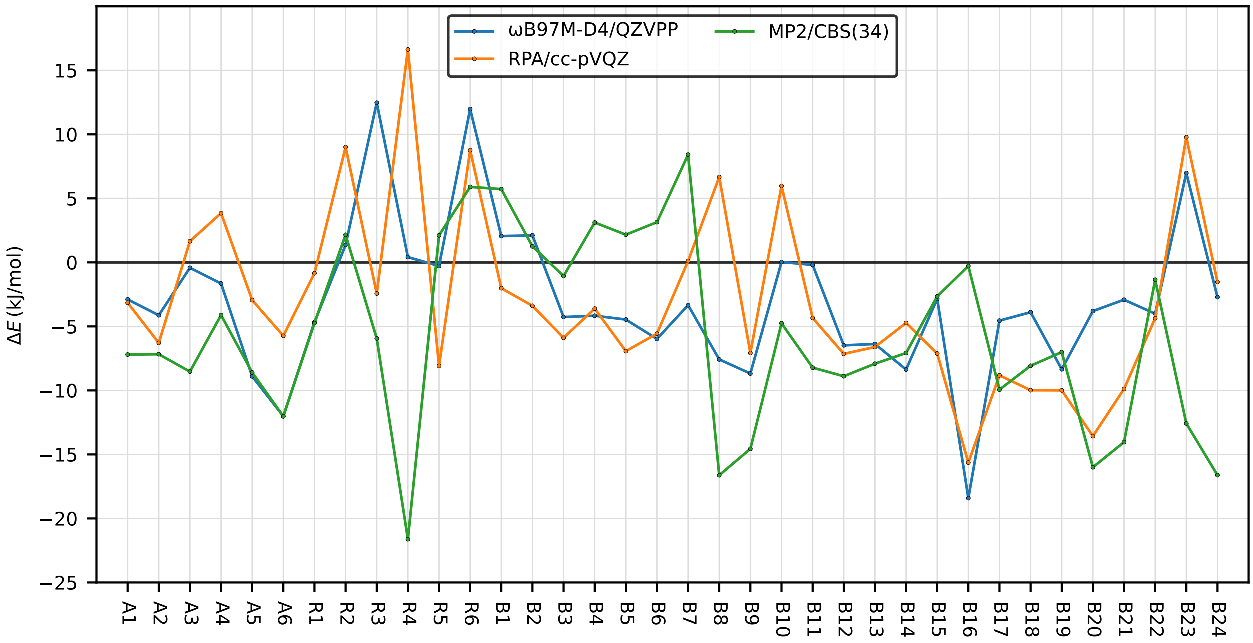

Figure S1. Analysis of the error relative to the reference method of the best-performing methods, RPA, MP2 and ωB97M-D4.

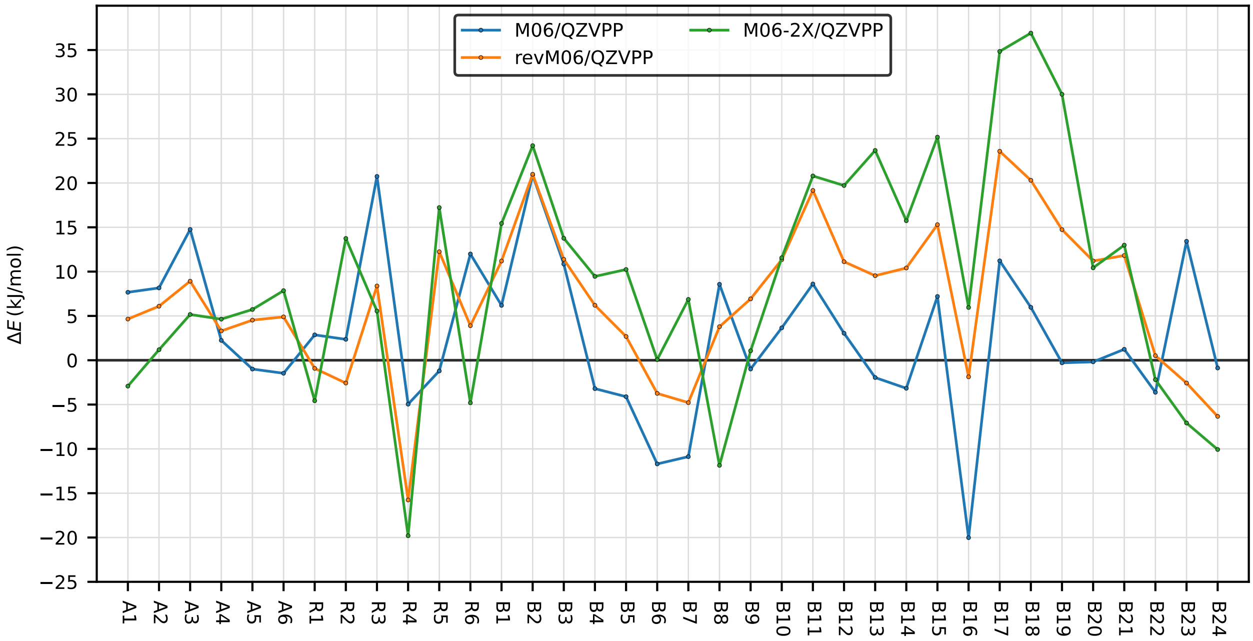

Figure S2. Analysis of the error relative to the reference method of Minnesota global hybrid functionals.

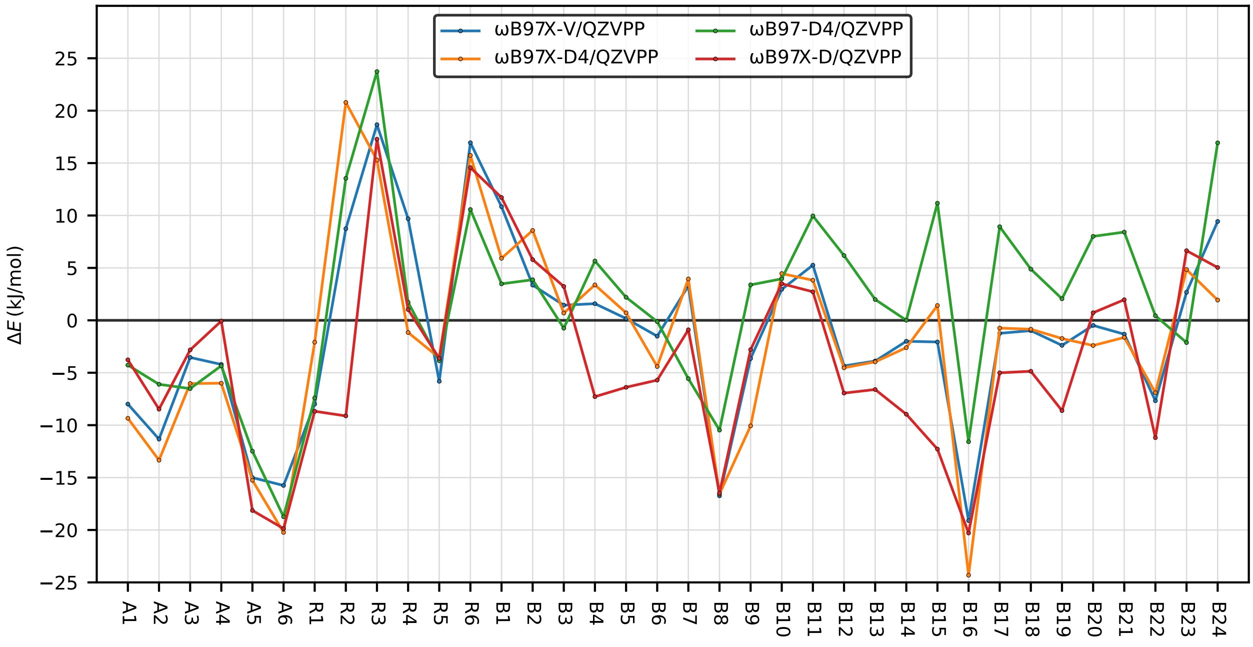

Figure S3. Analysis of the error relative to the reference method of selected range-separated hybrid functionals.

S4. Analysis of intrinsic barriers

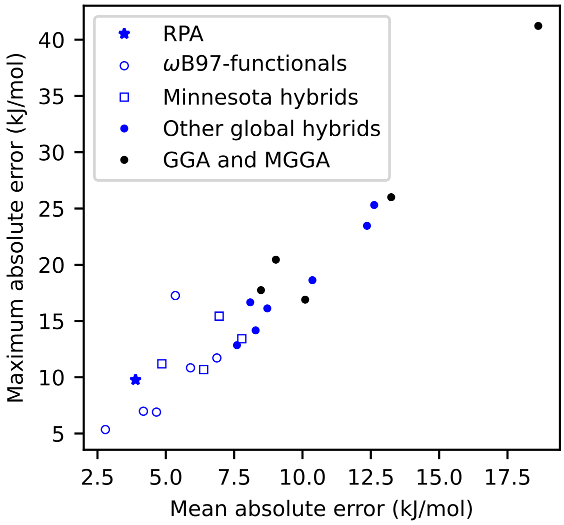

Figure S4. Analysis of intrinsic barriers only.

Table S3. Analysis of the error for barriers, separated by intrinsic (B1, B6, B7, B22, B23, B24) and apparent (all other) barriers. Mean absolute errors (MAE), mean signed errors (MSE) and maximum absolute errors (max.) in kJ/mol.

Method	intrinsic $\Delta E^{\ddagger}$			apparent $\Delta E^{\ddagger}$		
	MAE	MSE	max	MAE	MSE	max
RPA/cc-pVQZ	3.9	-0.6	9.8	7.6	-6.2	15.6
ωB97M-D4/QZVPP	4.2	-1.2	7.0	5.4	-5.2	18.4
ωB97M-V/QZVPP	2.8	-1.0	5.4	8.9	-8.9	26.8
M06/QZVPP	7.8	-1.2	13.4	6.4	2.6	20.9
revM06/QZVPP	4.9	-1.0	11.2	11.8	11.6	23.6
M06-2X/QZVPP	7.0	0.5	15.4	17.7	16.4	36.9
M06-SX/500eV	6.4	-3.1	10.7	13.9	13.7	27.2
r^{2}SCAN0-D4/QZVPP	7.6	2.0	12.9	10.2	-9.1	31.7
r^{2}SCAN0-rVV10/400eV	8.1	2.5	16.7	10.7	-9.0	41.2
r^{2}SCAN35-D4/QZVPP	8.7	5.6	16.1	5.2	0.2	25.6
r^{2}SCAN50-D4/QZVPP	12.4	10.7	23.5	15.9	14.2	26.2
ωB97X-D/QZVPP	6.9	0.9	11.7	6.9	-4.9	20.3
ωB97X-V/QZVPP	5.9	2.8	10.8	4.1	-2.4	19.1
ωB97X-D4/QZVPP	4.7	0.9	6.9	5.1	-2.6	24.3
HSE06-D3/400eV	10.4	1.9	18.6	10.4	-8.8	37.8
HSE38-D3/400eV	12.6	8.0	25.3	7.1	2.7	30.9
PBE0-D3/QZVPP	8.3	-0.6	14.2	17.7	-17.4	42.6
M06-L/QZVPP	9.0	-5.8	20.5	14.6	-12.1	40.2
revM06-L/QZVPP	8.5	6.1	17.7	18.1	16.6	34.4
B97M-V/QZVPP	10.1	-2.9	16.9	16.4	-16.4	31.4
r^{2}SCAN-D4/QZVPP	13.2	-8.2	26.0	33.0	-33.0	49.7
PBE-D3/def2-QZVPP	18.6	-13.5	41.2	46.3	-46.3	75.5
ωB97-D4/500eV	5.3	1.5	17.3	6.0	3.2	11.8

S5. Analysis of basis set convergence for 2T cluster models

Adsorption of methanol (adsorption A1) and SMS-formation (barrier B1) were studied for a 2T-model (clean: $(OH)_3Si{-}OH{-}Al(OH)_3$). All structures were fully optimized with PBE-D3/def2-SV(P). RI-MP2 and RI-RPA calculations were both carried out with TURBOMOLE. These small models give reaction energies and barriers that are typically far off from realistic models. However, they can serve to give an estimate of basis set convergence. Figures S2 to S5 show the results, for overall MP2 (RPA) and the separate contributions of HF and correlation. For adsorption, values with and without counterpoise correction (CP) are shown. For both MP2 and RPA, non CP-corrected results overbind and CP-results underbind, due to the contribution from the correlation energy. For MP2, the non-CP CBS-extrapolated results are indicated and can be seen to be good estimates of the CBS-limit. The most relevant adsorption energies for RPA are (Fig S3a), in kJ/mol): pVQZ: -64, pV6Z: -60 and CP-pV6Z: -57. The error of RPA/pVQZ is therefore about 5 kJ/mol.

Basis set convergence of the intrinsic SMS-formation barrier is much better as it is free of BSSE. Here, both MP2/CBS(34) and RPA/pVQZ give results that differ by less than 1 kJ/mol from the respective value obtained with the pV6Z basis set.

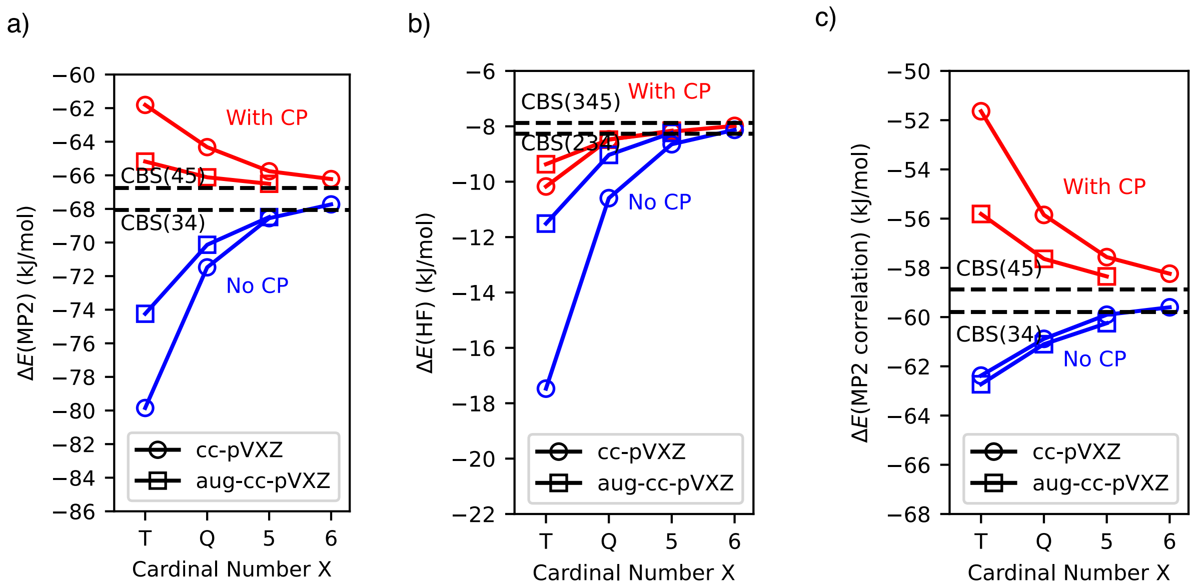

Figure S5. Convergence of MP2 for adsorption of MeOH on a 2T cluster model.

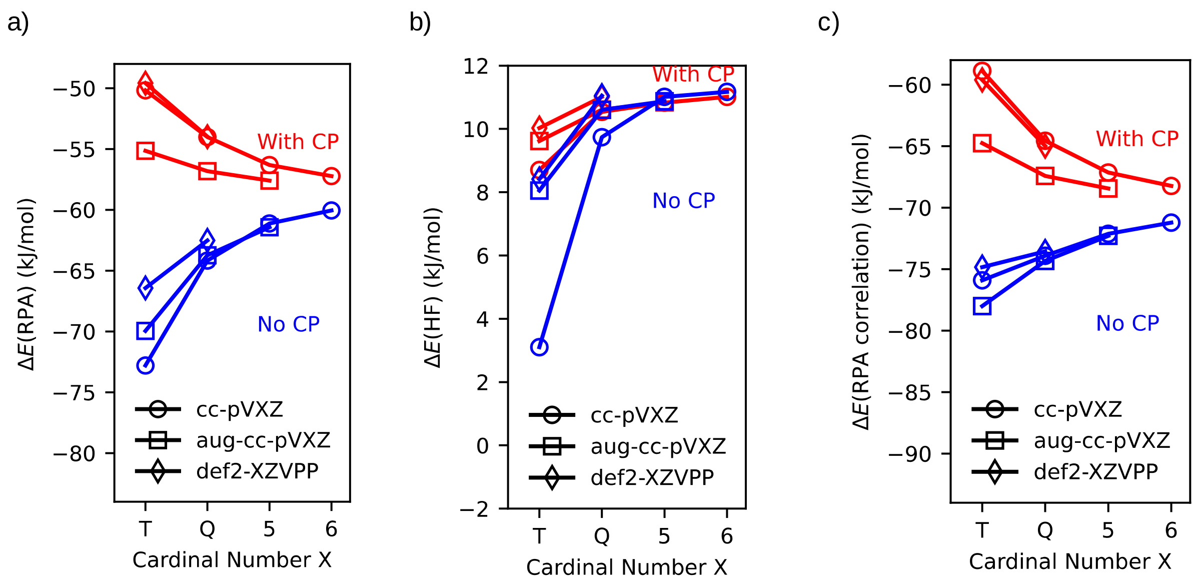

Figure S6. Convergence of RPA for adsorption of MeOH on a 2T cluster model.

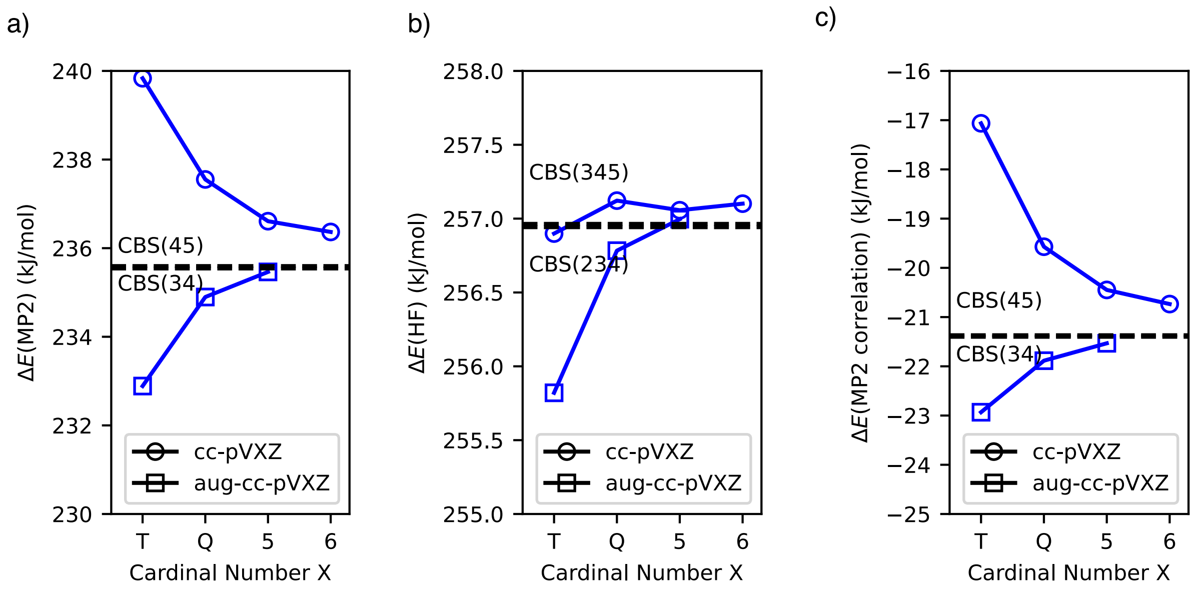

Figure S7. Convergence of MP2 for SMS formation on a 2T cluster model, given relative to adsorbed MeOH.

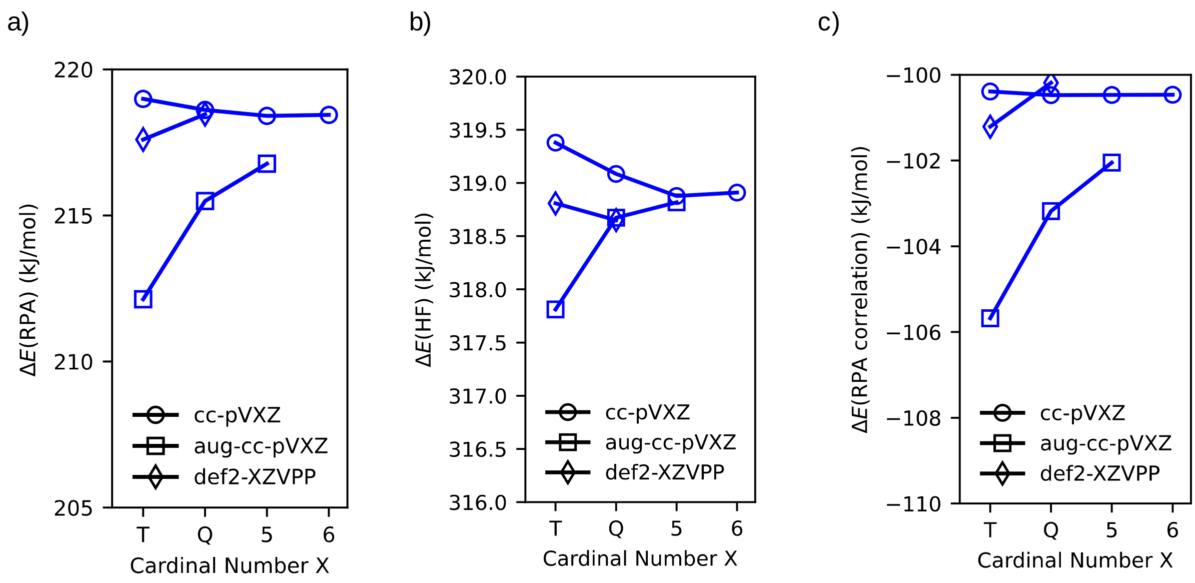

Figure S8. Convergence of RPA for SMS formation on a 2T cluster model, given relative to adsorbed MeOH.

S6. Effect of geometry optimization using a hybrid functional

The limitation of using only single-point calculations is investigated here for four reactions. Results obtained with HSE06-D3 are compared both fully optimized structures and as single points on top of PBE-D3 optimized structures for initial states, transition states and for scans along the reaction coordinates. The results are shown in Figs S9-S12. Table S4 lists the barriers.

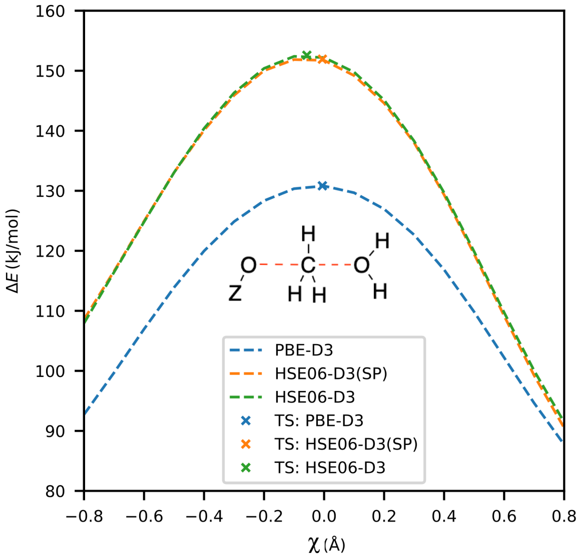

Figure S9. PBE-D3 energies and HSE06-D3 energies (single point computations at PBE-D3 optimized structures) for the reaction B1 (SMS and methanol to FA and methane) using constrained values for the difference of atom distances: χ = d(H_2O – CH_3) - d(ZO – CH_3). Transition states obtained with the ARPESS method are also depicted.

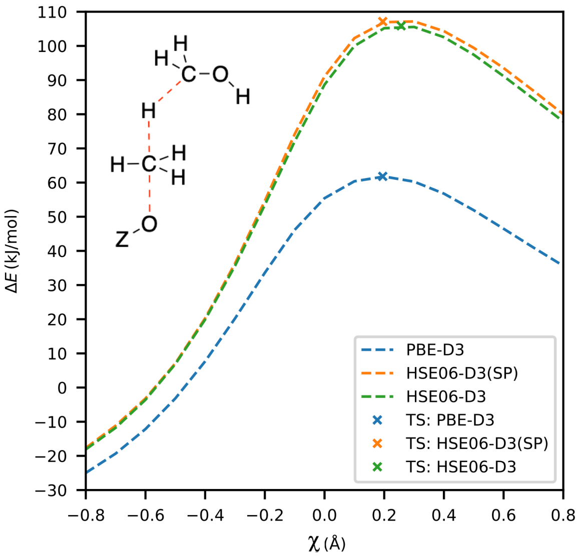

Figure S10. PBE-D3 energies and HSE06-D3 energies (single point computations at PBE-D3 optimized structures) for the reaction B4 (SMS and methanol to FA and methane) using constrained values for the difference of atom distances: χ = 0.5d(ZO – CH_3) + 0.5d(H – CH_2OH) - d(H_3C – H). Transition states obtained with the ARPESS method are also depicted.

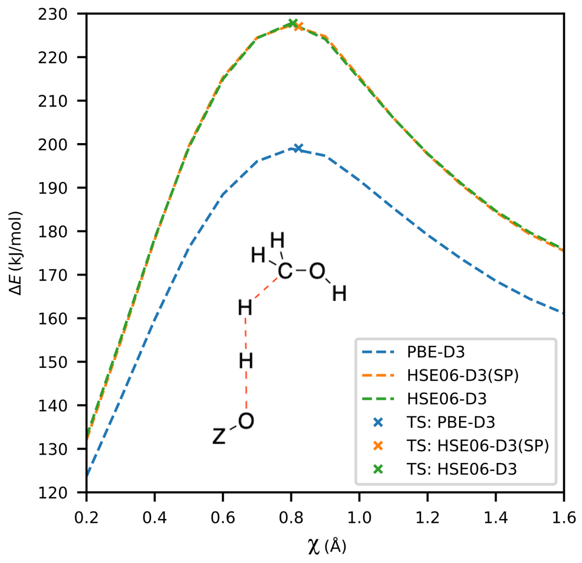

Figure S11. PBE-D3 energies and HSE06-D3 energies (single point computations at PBE-D3 optimized structures) for the reaction B6 (SMS and methanol to FA and methane) using constrained values for the difference of atom distances: χ = 0.5d(ZO − H) + 0.5d(H − CH_2OH) - d(H − H). Transition states obtained with the ARPESS method are also depicted.

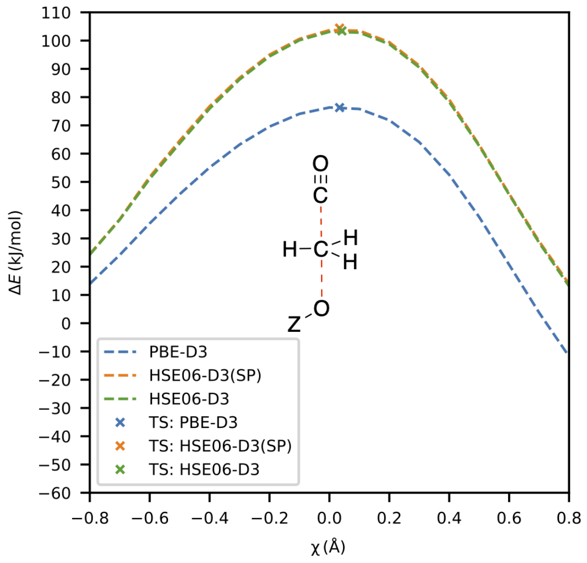

Figure S12. PBE-D3 energies and HSE06-D3 energies (single point computations at PBE-D3 optimized structures) for the reaction B10 (SMS and methanol to FA and methane) using constrained values for the difference of atom distances: χ = d(ZO − CH_3) - d(OC − CH_3). Transition states obtained with the ARPESS method are also depicted.

Table S4. Barriers obtained with fully optimized HSE06-D3 calculations and as single points on PBE-D3 calculations (HSE06-D3//PBE-D3). Values in kJ/mol.

	HSE06-D3(single-point)	HSE06-D3
TS B1	151.9	152.5
TS B4	107.2	105.9
TS B6	227.0	227.7
TS B10	104.4	103.5

S7. DLPNO-CCSD(T1) vs. DLPNO-CCSD(T0)

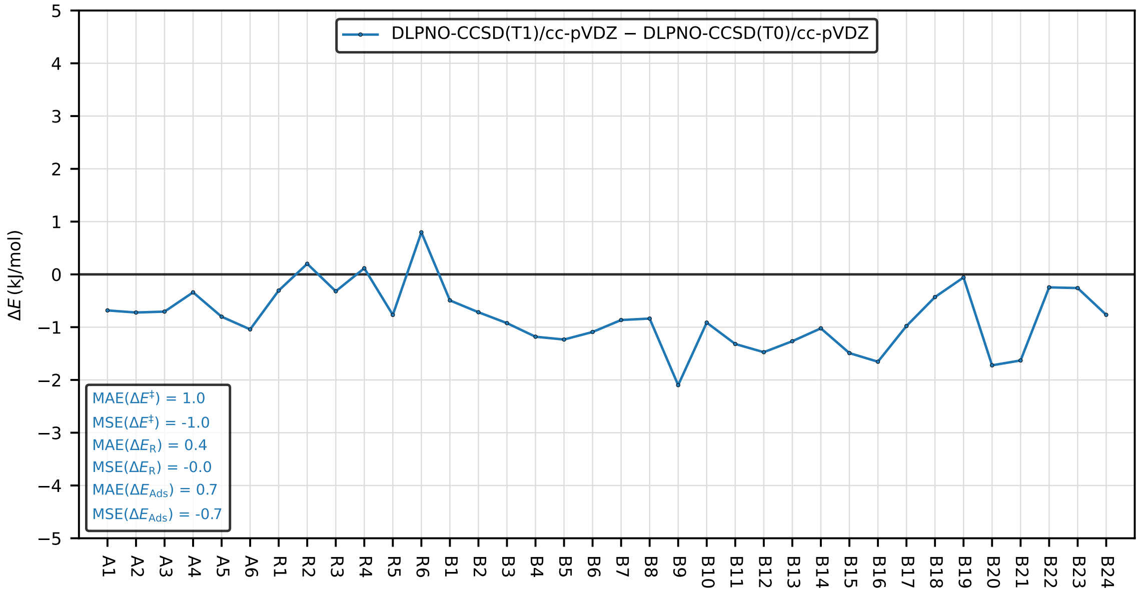

Figure S13. Difference between the results obtained with DLPNO-CCSD(T1)/cc-pVDZ and DLPNO-CCSD(T0)/cc-pVDZ
